# Membrane Charge Drives the Aggregation of TDP-43 Pathological Fragments

**DOI:** 10.1021/jacs.5c00594

**Published:** 2025-04-08

**Authors:** Giacomo Corucci, Devkee M. Vadukul, Nicolò Paracini, Valérie Laux, Krishna C. Batchu, Francesco A. Aprile, Annalisa Pastore

**Affiliations:** 1 Department of Chemistry, Molecular Sciences Research Hub, Imperial College London, London W12 0BZ, U.K.; 2 Institut Laue Langevin, Avenue des Martyrs 71, Grenoble 38000, France; 3 Data Management and Software Centre, European Spallation Source ERIC, Asmussens Allé 305, Lyngby 2800, Denmark; 4 Institute of Chemical Biology, Molecular Sciences Research Hub, Imperial College London, London W12 0BZ, U.K.; 5 Institute of Brain Sciences, Burlington Danes, The Hammersmith Hospital, Du Cane Road, London W12 0NN, U.K.; 6 The Wohl Institute, King’s College London, 5 Cutcombe Rd, London SW59RT, U.K.; 7 Elettra Sincrotrone Trieste, s.s. 14 km 163,500, Area Science Park, Basovizza, Trieste 34149, Italy

## Abstract

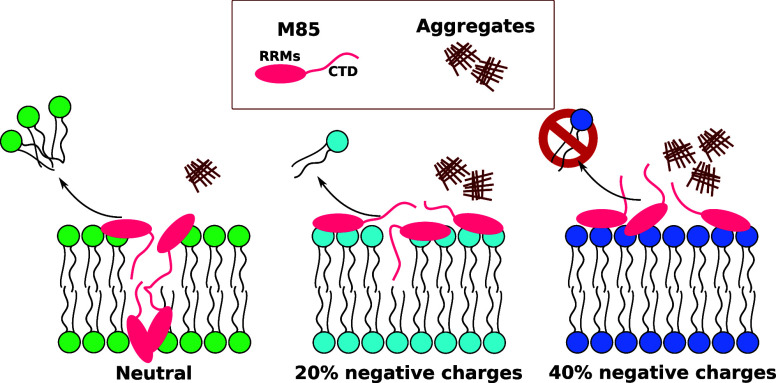

TDP-43 protein is an RNA-binding protein linked to amyotrophic lateral sclerosis, frontotemporal dementia, and Alzheimer disease. While normally a protein that shuttles between the nucleus and cytoplasm, TDP-43 has recently been found also in extracellular vesicles. These are an important medium for cell–cell communication that allows the transfer of lipids, proteins, and genetic material among cells. An increasing concern in neurodegenerative diseases, however, is the possibility that extracellular vesicles can also provide an effective way to spread misfolded proteins that could “infect” other cells according to a “prion-like” mechanism. To characterize the interaction of TDP-43 with lipid membranes, we carried out a systematic biophysical study using a TDP-43 fragment lacking the first 84 N-terminal residues, called M85, and synthetic model phospholipid membranes. We utilized standard techniques, such as fluorescence and microscopy, complemented by neutron reflectivity measurements. Our results show that lipid charge affects the modality by which M85 interacts with membranes: a higher negative charge induces the protein to bind to the bilayer surface, promoting protein aggregation and decreasing lipid bilayer damage that this interaction causes. Thus, we speculate that the M85-lipid membrane interaction could play an important and previously undefined role in TDP-43-related neurodegenerative diseases.

## Introduction

Transactivation response DNA-binding protein 43 (TDP-43) is one of the main players in two distinct but etiologically related diseases, amyotrophic lateral sclerosis (ALS) and frontotemporal dementia (FTD),^1−4^ through formation of ubiquitin-positive tau-negative inclusions found in the affected neurons of a large number of FTD and of almost all ALS patients.^2,5^ Mutations in several other genes are also independently associated with these two pathologies leading to a common phenotype.^6,7^ Encoded by the TARDBP gene, TDP-43 is a modular protein of 414 residues^8^ containing an N-terminal ubiquitin-like domain, a nuclear export signal,^9^ two RNA/DNA-recognition motifs (RRM1 and RRM2 spanning residues 106–176 and 191–262, respectively), and a C-terminal glycine-rich region (TDP-43 274–414) that was classified as a prion-like domain on the basis of characteristic sequence features.^10^ TDP-43 aggregates are also found in the brain of Alzheimer disease (AD) and in patients with forms of Parkinson.^11^ In healthy cells, TDP-43 acts as a multifunctional RNA-binding protein mainly localized in the nucleus where it regulates RNA transcription and splicing.^12,13^ Under pathological conditions, TDP-43 mislocalizes and forms cytoplasmic aggregates that are a hallmark of both ALS and FTD.^14^

In addition to these two main compartments, increasing evidence suggests that direct interaction between TDP-43 and membranes plays a role in pathological development.^15^*In vitro* studies have, for instance, shown that TDP-43 has indeed prion-like properties and circumstantial evidence has suggested that disease spreading of intracellular TDP-43 protein aggregates could be facilitated by extracellular vesicles (EVs).^16,17^ These are lipid bilayer particles ranging from 30 nm to 5 μm in size^18,19^ that are naturally secreted into the extracellular space by almost all types of cells.^20,21^ EVs are important in a vast number of cell-to-cell communication processes to both neighboring and distant cells through the transfer of lipids, proteins, and genetic material.^22^ They are also important in cancer metastasis and are extensively being used clinically as carriers of biomarkers for diagnostics and drug delivery.^23,24^ In the central nervous system, EVs can contribute to both physiological and pathological processes. In neurodegenerative diseases, the affected neurons can release misfolded proteins in EVs as a protective mechanism for the secreting cell, but this secretion can in turn be highly effective in spreading misfolded material, such as aggregates of amyloid-β (Aβ), tau, α-synuclein, prions, and many other examples, which is thought to be able to “infect” other cells according to a “prion-like” mechanism.^25^ The role of EVs in contributing to the spreading of toxic material has attracted particular interest in studies of various forms of dementia in which EVs seem to exacerbate neurodegeneration and accelerate disease progression.^15,26^ On the other hand, EVs are promising candidates as new diagnostic and therapeutic tools due to their innate ability to traverse the blood–brain barrier and their ubiquitous nature.^27^ Thus, understanding how proteins interact with lipid membranes has become an essential step to comprehend disease mechanisms in neurodegeneration and be able to use the interaction as a marker for disease progression. Experiments in SH-SY5Y cells expressing TDP-43 have shown that, when treated with insoluble TDP-43 inclusions isolated from ALS patients’ brain, full-length TDP-43 significantly increases in the EV fraction of these cells.^17^ Propagation of TDP-43 inclusions was observed by addition of EVs from the cerebrospinal fluid of ALS/FTD patients to glioblastoma or Neuro2a cells.^2,28,29^ Vesicular TDP-43 is taken up by recipient cells preferentially in comparison to free TDP-43, resulting in increased toxicity.^30,31^ The involvement of EVs in the transport of pathologic TDP-43 was further confirmed by a recent study^32^ in which the authors observed EVs containing TDP-43 as well as its disease-associated fragments, TDP-35, and TDP-25, that are N-terminally truncated.^33,34^ Observation of TDP-43 fragments in vesicles is particularly interesting since, despite the initial assumption that intramembrane cleavage (cleavage process occurring within a lipid membrane) could be an abnormal event exclusively associated with disease, intramembrane cleavage is now considered a normal proteolytic pathway that mediates the generation of various functional variants.^35−37^

Despite extensive research conducted on the involvement of TDP-43 in ALS and FTD, investigations on the contribution of lipids in general and of EVs in particular to TDP-43 aggregation have only begun in recent years. Yet, lipids play a dominant role in the aggregations of several proteins related to neurodegeneration.^38,39^

To further understand the role of the interactions of TDP-43 with lipid membranes, we present here how the N-terminally truncated 35 kDa fragment of TDP-43 starting at M85, hereafter indicated as “M85”, interacts with lipids using large unilamellar vesicles (LUVs), supported lipid bilayers (SLBs), and lipid monolayers at the air/water interface. We mainly used for our studies model synthetic membranes assembled by robust protocols widely described in the literature to ensure full control of our system.^40^ We utilized state-of-art biophysical techniques, such as fluorescence, microscopy, and neutron reflectivity measurements, that are particularly effective to assess protein–lipid interactions. In our experiments, we systematically varied the lipid composition of the membranes to probe how lipid charge and charge density affect M85-membrane interactions as well as the conformation and stability of both the protein and lipid bilayer. Our results confirm an interaction and show that the lipid charge affects the modality by which M85 interacts with the membrane: the protein binds the bilayer surface and inserts into it when the lipids are zwitterionic and thus neutral. At increasing membrane charge, the protein gets progressively less bound but tends to increase its aggregation tendency. Thus, we speculate that the M85-lipid membrane interaction could play an important and previously uncharacterized role in TDP-43-associated neurodegenerative diseases.

## Materials and Methods

### Chemicals and Reagents

Synthetic lipids (POPC: 1-palmitoyl-2-oleoyl-glycero-3-phosphocholine, POPS: 1-palmitoyl-2-oleoyl-*sn*-glycero-3-phospho-l-serine, and POPA: 1-palmitoyl-2-oleoyl-*sn*-glycero-3-phosphate) were purchased from Avanti Polar Lipids with a purity of >99%. HPLC-grade chloroform and methanol were purchased from Sigma-Aldrich. D_2_O was purchased from Sigma-Aldrich. *Pichia pastoris*-deuterated polar extract was produced by the ILL Lipid Laboratory (L-lab and D-lab) according to previous protocols.^41,42^

### Expression and Purification of the M85 Variant

M85 comprises residues 85–414 of full-length TDP-43 (Figure S1). M85 was expressed in the BL21 DE3 *Escherichia coli* cell strain using the pET His6 Sumo TEV expression vector as fusion protein composed of an N-terminal His tag, a SUMO tag, and the M85 protein sequence. Cells were allowed to grow in 2.5 L flasks with 1 L of LB medium (Lennox) at 37 °C until an optical density of 0.6 measured at 600 nm was reached. Flasks were placed in the cold room (4 °C) for 30 min to cool down the grown cells. Protein expression was induced using 1 mM IPTG at 18 °C with shaking at 200 rpm overnight. Cells were collected through centrifugation at 4500*g* for 30 min at 4 °C, and the pellet was stored at −20 °C. The majority of the following protocol was optimized for the full-length TDP-43 purification by the Hasnain team.^43^ The frozen pellet was thawed in ice, resuspended in Milli-Q water, DNase 1 μg/mL, RNase 1 μg/mL, and PMSF 5 mM, and sonicated for 5 min (total ON time) with pulses (1 s ON and 3 s OFF) with an amplitude of 35%. The lysate was then centrifuged at 35,000*g* for 1 h at 4 °C to remove cell debris. A lysis buffer containing phosphate buffer (50 mM, pH 8.0) with 0.2% sarkosyl was added to the supernatant at a ratio of 1:1. The sample was filtered using 0.45 μm syringe filters before Ni affinity chromatography. The first Ni affinity chromatography (Cytiva HisTrap FF column) was performed, and the flowthrough was discarded. The elution step was carried out using a gradient (0 to 100% in 15 CV where the protein was eluted at around 30% elution buffer). The binding buffer composition was 50 mM phosphate buffer pH 8.0, 0.2% sarkosyl, and 300 mM NaCl, whereas the elution buffer was 50 mM phosphate buffer pH 8.0, 300 mM NaCl, and 500 mM imidazole. TEV protease (purified in-house) was added to the eluted proteins at a 3:1 ratio (TEV:M85) while dialyzing against 50 mM phosphate buffer pH 8.0, 300 mM NaCl, 2% glycerol, and 5 mM DTT overnight in the cold room. The next day, a second Ni affinity chromatography was performed to purify the cleaved protein. The flowthrough containing the protein was dialyzed against 50 mM phosphate buffer, pH 8.0, and 6.5% glycerol. A cationic exchange chromatography (HiTrap Q HP 1 mL) was finally performed to remove the remaining contaminants. The protein, during this last purification step, was collected in the flowthrough. A third dialysis was next performed against the same buffer, and the protein was aliquoted, flash-frozen using liquid nitrogen, and conserved at −80 °C. All chromatography steps were carried out on the AKTA Pure system (Cytiva). The protein preparations were analyzed using an Agilent electro-spray-ionization time-of-flight mass spectrometer (ESI-TOF) in positive mode.

### Western and Dot Blotting

Western blots were performed using an iBlot 2 Invitrogen Thermo Fisher Scientific for semiwet transfer in a nitrocellulose membrane. Dot blots were performed using a nitrocellulose membrane, where samples were loaded in duplicates. A primary monoclonal antibody anti-TDP-43 (dilution 1:1000) (from Proteintech) and monoclonal anti-His (dilution 1:1000) were used with an antimouse as secondary fluorescent Alexa fluor 647 antibody (dilution 1:2000). A Typhoon FLA 9500 imager (GE Healthcare) was used to scan membranes, and ImageJ software was used for integrating the bands/dots from the acquisitions. Errors in the integrations were calculated as the standard deviation from the replicates.

### Lipid Vesicle Preparation

Phospholipids, conserved in a solvent mixture of chloroform:methanol 9:1 ratio at −20 °C in glass vials, were mixed to form the desired ratio of phospholipid mixtures. The solvent was next evaporated from the sample using a nitrogen stream, and the resulting lipid film on the bottom of the glass vial was then stored under vacuum overnight to ensure complete evaporation of the solvent. The film was then rehydrated at room temperature using different buffers, depending on experimental needs. Upon addition of the buffer, the vial was filled with nitrogen to remove the excess air and avoid lipid oxidation. The solution was vortexed until the film was completely resuspended, forming an opaque solution. Tip sonication was next carried out (5 min, 1 s on and 5 s off at 20% amplitude in ice to avoid overheating) to break down large or multilamellar vesicles, which were unsuited for the experiments presented in this work.

### Vesicle Preparation for ThT Fluorescence Assays and Transmission Electron Microscopy

The lipid film formed (usually 1 mg total amount per vial) was rehydrated using 50 mM phosphate buffer at pH 8, and extrusion using an Avanti Mini Extruder with 100 nm cutoff membranes was performed after sonication to obtain uniform and monodispersed vesicle solutions (Large Unilamellar Vesicles—LUVs). The process was performed 35 times per lipid sample at ambient temperature (around 22 °C).

### Vesicle Preparation and Vesicle Fusion Protocol to Obtain Supported Lipid Bilayers for Neutron Reflectivity Measurements

Lipid films (1 mg per vial) were rehydrated with a solution of 0.5 M NaCl in Milli-Q water, and after sonication, the solution was immediately utilized for vesicle fusion to form SLBs. Vesicle fusion was carried out by equilibrating the neutron reflectivity cell with 0.5 M NaCl; the vesicle solution was then injected and incubated for 30 min to allow vesicles to interact with the silicon substrate. A total amount of 1.5 mL (with a lipid concentration of around 0.7 mg/mL) was enough to fill one cell. Next, Milli-Q water was injected into the cell to wash the nonbounded vesicles as well as to create an osmotic shock by replacing the salt solution with water. This last step allowed vesicles to open, forming a single lipid bilayer deposited on the silicon crystal inside the cell. Next, the water was replaced with the experiment buffer (50 mM phosphate buffer pH 8.0), and measurements were started.

### Transmission Electron Microscopy

Formvar/carbon-coated 300 mesh copper grids were utilized. Prior to utilization, the grids were glow-discharged to clean and obtain a hydrophobic surface. The sample (5 μL) was spotted in each grid, followed by 2 min of incubation. The excess was removed by using filter paper. Washing with Milli-Q water was carried out to remove the nonbounded particles. Grid staining was then performed by adding 5 μL of a solution of 2% w/v uranyl acetate for 2 min followed by Milli-Q water washing. Final grids were imaged on a T12 Spirit electron microscope (Thermo Fisher Scientific (FEI), Hillsboro, OR, USA).

### ThT Fluorescence Assays

Samples were prepared by adding ThT (10 μM final concentration), followed by gentle vortexing. Samples were then placed in a half-area 96-well nonbinding plate (Greiner Bio-One, Frickenhausen, Austria) in triplicates. ClarioStar Plus microplate reader (BMG LabTech, Ortenberg, Germany) was utilized for these measurements. The plate was incubated at 37 °C for the duration of the assay. The excitation and emission wavelengths were set to 440 and 480 nm, respectively, and fluorescence intensity measurements were taken using spiral averaging (3 mm diameter). The reading was taken every 5–10 min. Data were analyzed using an ad-hoc python program that subtracted the buffer and normalized the signal. Data were further analyzed using a simple sigmoidal function as shown in Equation S1 to be able to calculate the t50 of the aggregation profiles.

### Neutron Reflectivity Measurements

Neutron reflectivity experiments on SLBs at the solid/liquid interface were performed on FIGARO, a time-of-flight reflectometer available at the European Neutron Source of the Institute Laue-Langevin (ILL) in Grenoble (France).^44^ Two different angles of incidence Θ_1_ = 0.7° and Θ_2_= 3.0° were utilized to cover a momentum transfer (*q*_*z*_) range from about 8 × 10^–3^ to 0.25 Å^–1^, in combination with a wavelength range of 2–20 Å for both setups with a *q*_*z*_ resolution of 7% dλ/λ. The momentum transfer *q*_*z*_ is defined as shown in eq 1.
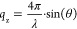
1where λ is the wavelength of the neutron beam. The reflectivity *R*(*q*_*z*_) is defined as the ratio of the reflected intensity over the incident beam intensity (direct beam) and was calculated using the software COSMOS. The background was measured on the left and right sides of the specular reflectivity signal, directly subtracted from it, and subsequently divided by the direct beam intensity measured using the same slit configuration in transmission mode through the silicon crystals.

The analysis of the reflectivity data was performed using an in-house developed code, CoruxFit, described in ref (41). Briefly, the SLBs were modeled as a series of layers called components in CoruxFit, with different chemical composition stacked along the *z*-axis with respect to the silicon crystal. The lipid bilayer was divided into three components (polar heads, hydrophobic chains, and polar heads again to account for the bilayer). The following components were used to describe the SLB: (1) bulk silicon, (2) silicon oxide layer, (3) inner polar heads layer, (4) hydrophobic chains, and (5) outer polar heads layer. A component volume fraction distribution was assigned to each of the identified components according to eq 2,
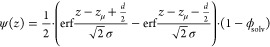
2where erf(*x*) is defined as follows:
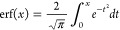
In eq 2, *z*_μ_ describes the position of the component along the *z*-axis, *d* describes its thickness, and σ is the roughness of the interface between two consecutive components. The first term of the error function erf of the equation describes where the component starts, whereas the second term of the equation describes where the component ends. For some of the components used, the first or the second term was set to zero to recreate a fraction volume distribution with an undefined thickness (for instance, for the silicon support, the first term was set to zero). Each component is scaled by the amount of buffer in that layer through the parameter ϕ_solv_. The water profile along *z* axis is calculated so that adding up every component yields 1 according to eq 3,
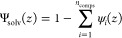
3where Ψ_solv_(*z*) is the fraction volume distribution of water along the *z*-axis and *n*_comps_ is the total number of components constituting the model. The model considers the phospholipid molecules as cylinders shaped, where the topological area per lipid is the constrain parameter for the thickness calculations of each component of the bilayer according to the equation:
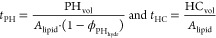
4where *t*_PH_ and *t*_HC_ are the thicknesses of the layers for the polar heads (PH) and hydrophobic chains (HC), respectively, PH_vol_ and HC_vol_ are the polar head and hydrophobic chain volumes, the *A*_lipid_ is the topological area per lipid, and finally, ϕ_PH_hydr__ is the hydration water of the polar head region. This last parameter is used as ϕ_solv_ in eq 2 to scale the fraction volume of the PH region for the amount of hydration water. Another fundamental parameter for the model utilized is ϕ_defects_, which is used as ϕ_solv_ for both HC and PH region, representing the amount of water through the whole lipid bilayer (defects of the lipid bilayer). In fact, the final amount of water in the PH region is calculated as ϕ_defects_ + ϕ_PH_hydr__, resulting in the parameter ϕ_PH_ that is shown in the tables. The HC region volumes were calculated using the following chemical groups volumes: CH_2_ = 27.5 Å^3^, CH_3_ = 55.1 Å^3^, and CH = 22.2 Å^3^ for lipids in fluid phase.^45^ The volumes for the different polar heads were calculated from their relative mass densities (PC = 318 Å^3^, PS = 263 Å^3^, and PA = 250 Å^3^). The scattering length densities (SLDs) of each component were calculated summing each scattering length of each atom constituting the component divided by the volume of the component. The fitted parameters for the bilayer analysis were: (1) *A*_lipid_; (2) ϕ_defects_; (3) ϕ_PH_hydr__; and (4) ϕ_ox_, where ϕ_ox_ is the ϕ_solv_ of the fraction volume distribution for the oxide layer of the silicon crystal (amount of water in the oxide layer). For the bilayer measurements, the parameters describing the oxide layer (such as thickness and roughness) were previously analyzed using the data collected on the bare surfaces in D_2_O contrast and were kept constant during the fit of the bilayer parameters (except the ϕ_ox_ values that can be different between bare and occupied surfaces). This approach decreases the number of fitted parameters and leads to an improvement in the accuracy of the final result. The interface roughness (σ) between each component describing the lipid bilayer was kept constant at a value of 3.0 Å. For the data analysis of the interaction M85–lipid bilayer, another component was added to the model using eq 2. M85 protein was approximate to a cylinder with a fixed volume. The height of this geometrical cylinder (that is defined as the thickness of this component) was calculated by dividing the volume with its topological area (that was adjusted during the fit of the curves). During the fit process, the *z* position of the component (*z*_μ_) was set free to vary to assess the best position of the protein along the *z*-axis of the lipid bilayer. The molecular volume and SLD of the M85 were estimated using the ISIS bioinformatic tool (Biomolecular Scattering Length Density Calculator (http://psldc.isis.rl.ac.uk/Psldc/)) and resulted to be 42,120.8 Å^3^ of volume and 2.00 × 10^–6^ and 3.32 × 10^–6^ Å^–2^ of SLD in hydrogenous and deuterated environments (H_2_O and D_2_O), respectively. The SLD of the protein was linearly calculated by CoruxFit from these two values, depending on the amount of D_2_O utilized for the measurements of the sample.

The total SLD profile of the sample was calculated by multiplying each component of the final model by the corresponding SLD values and summing them together to obtain the complete SLD profile. The theoretical reflectivity curve was calculated using the Parrat algorithm^46^ from the SLD profiles. CoruxFit minimizes the χ^2^ first by the simulated annealing method to test the whole parameter space and to avoid local minima, and then using the steepest descent algorithm (link to the python library: https://docs.scipy.org/doc/scipy/reference/generated/scipy.optimize.least_squares.html), that cools down the system finding the best parameter combination with the lowest χ^2^. Errors on the fitted parameters were calculated as the square root of the diagonal elements in the inverse Hessian matrix multiplied by final χ^2^. The errors for the derived parameters, such as the bilayer thicknesses, were calculated assuming standard error propagation. The *q* resolution of the instrument was also taken into account during the calculations of the theoretical reflectivity profiles to smear features accordingly.

Measurements of the SLBs prior to and after M85 mixture injection were performed in two or three contrasts, to lower the ambiguity of the neutron reflectivity analysis. The three contrasts utilized in this study are H-buffer (phosphate buffer solubilized in Milli-Q water), D-buffer (in pure D_2_O), and the Silicon Matched Water (SMW, that is a mixture of the H and D-buffers at a ratio of 37%D and 63%H, matching the SLD of the silicon crystal). The kinetics after the M85 mixture injection were performed in H-buffer since this buffer was used to store the protein preparation.

The integrated reflectivity intensities for the kinetics measurements with neutron reflectivity were calculated summing all the data points between 8 × 10^–3^ and 0.1 Å^–1^ for each sample. Errors of the integrated reflectivity intensity were calculated using the standard error propagation of each experimental point of the reflectivity profile, as also described in ref (47).

### Reflectivity Substrates Description and Cleaning Procedures

The silicon crystal utilized for the measurements was a 111 single crystal 8 × 5 cm^2^ with a thickness of 1.5 or 2 cm, polished on one face with a roughness below 5 Å. Crystals were cleaned with chloroform, acetone, ethanol, and Milli-Q water in this order. Each washing step was performed in a bath sonicator for 30 min to increase the cleaning ability of the solvents. Plasma cleaning was performed just before the usage to clean the polished surface of the crystals. This process allows this area to become more hydrophilic favoring the formation of the SLB by enabling its interaction with the polar head side of the lipid.

### Monolayer Formation and Ellipsometry Analysis

Monolayer experiments were performed using a KIBRON G1 Langmuir trough. Phosphate buffer at pH 8.0 (50 mM) was used as the bulk solution, above which the lipid monolayer was formed. An amount of 45 μL of 0.5 mg/mL lipid solution in chloroform:methanol 9:1 was spread on the air/water interface and left for 30 min to allow the solvent to evaporate. The monolayer was compressed (with the barrier at a speed of 10 mm/min) to 20 mN/m surface pressure and held in place for stabilization. Once the pressure was stabilized, the protein was injected into the bulk solution, and the measurements of both surface pressure and ellipsometry were started. Ellipsometry measurements were performed using a Beaglehole Picometer Light ellipsometer (New Zeland) equipped with a He–Ne laser at a wavelength of 632.8 nm. The incident angle to the surface of the air/water interface was set to 52° (near the Brewster angle of the air/water interface that is 53°). Data were acquired every 5 s after the injection of the protein into the bulk solution.

## Results

### Rational Selection of Lipid Composition of Mono and Bilayers

Selecting the appropriate lipid composition can be challenging. While human neuron EVs represent the natural environment for TDP-43, studying protein–lipid interactions requires techniques that go beyond replicating physiological conditions to explore broader biophysical properties, similarly to protein stability studies, which utilize extreme heat, pressure, or pH conditions to understand protein properties. This situation is like that encountered in studies of protein stability: while most proteins would never experience extreme heat, pressure, or pH conditions, it is customary to use thermal, pressure, and pH scans to understand regions of the phase diagrams of the protein in its full potential.

**Figure 1:**
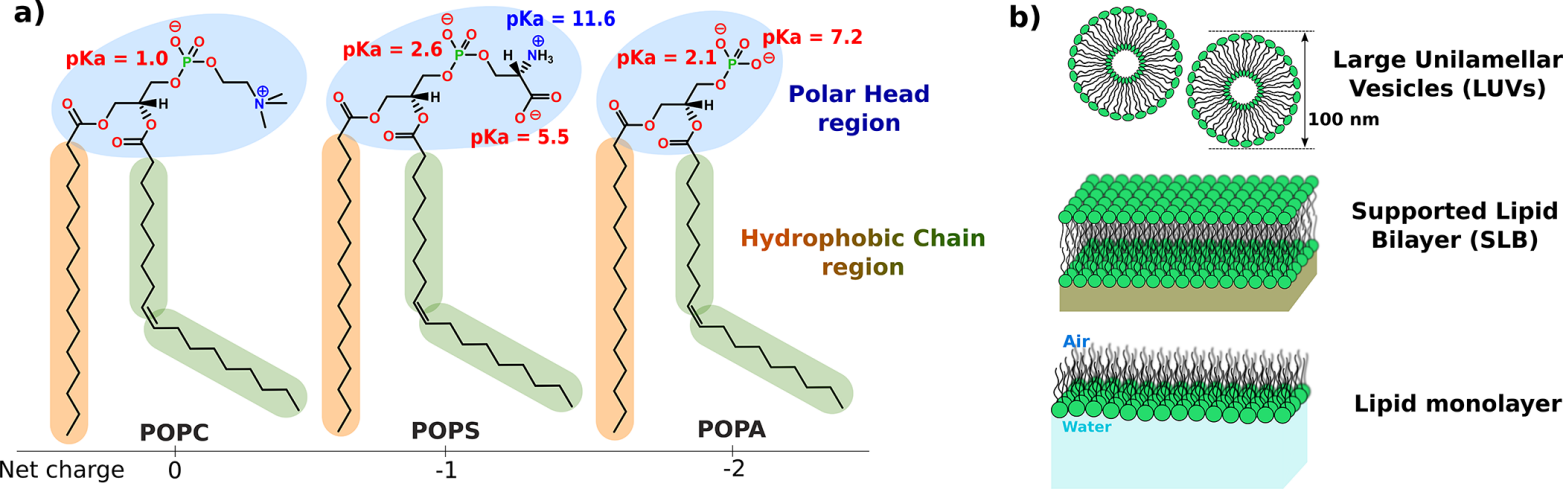
Molecular structure of the synthetic lipids and model membranes used. (a) Common chains were made of palmitoyl and oleyl fatty acids (PO) account for the acyl chain composition, and they differ from the number of carbons in the acyl chain (16 and 18, respectively) and the number of double bonds (none and 1, respectively). This composition mimics the average composition of eukaryotic cells. The polar head region is different between the three lipids selected, where PC (phosphatidyl choline) is a neutral zwitterionic polar head, and PS (phosphatidyl serine) is negatively charged, as well as PA (phosphatidic acid) with a higher negative charge density due to its small volume and its negative charge of −2 (at pH 8). (b) Schematic representation of the three model membranes utilized in this work.

In the present study, we decided to use membranes composed of synthetic phospholipids to systematically explore the role of their charges on M85-membrane interactions. This is an essential aspect that can provide valuable insights into the nature and mechanisms of these interactions. We thus adopted synthetic membranes composed of commonly used natural phospholipids: POPC, POPS, and POPA (Figure 1a). These phospholipids are progressively negative, displaying a net charge of 0, −1, and −2, respectively. In addition to possessing a higher negative charge (−2) at our pH (8.0), POPA has a smaller polar head, leading to a unique high charge density.^48^

In our experiments, we used monolayers (for preliminary ellipsometry measurements), LUVs for ThT assays and TEM measurements, and SLBs for neutron reflectivity measurements (Figure 1b). The latter are cell-membrane-mimicking flat platforms that are deposited onto solid surfaces and are suitable for neutron reflectivity measurements that yield the structure of the protein lipid complex along the normal to the interface. In addition, we used deuterated polar lipid extracts (named *Dpol*) from the *Pichia pastoris* yeast in a pilot experiment aimed at demonstrating the feasibility and as a matter of comparison with the better defined synthetic membranes. The advantage of this lipid mixture is that, coming from a eukaryotic organism, it is naturally closer to the neuron membrane wall than synthetic mixtures while maintaining the system rather simple. The extracts contain phosphatidylcholine (PC, 56% mol/mol), phosphatidylserine (PS, 9%), phosphatidylethanolamine (PE, 24%), phosphatidylinositol (PI, 7%), phosphatidylglycerol (PG, 3%), and cardiolipin (CL, 3%),^41,42^ with a total negative charge of around 25% as compared to pure POPS or 12.5% of pure POPA (percentage refers indeed to the amount of negative charges over the amount of phospholipids). The fatty acyl composition in EVs is enriched in lipids of a higher degree of polyunsaturation, but overall, the acyl chain profile in yeast more closely resembles that of the mammalian cells. Additionally, *Pichia pastoris* can be grown in a perdeuterated environment. The possibility to have fully perdeuterated lipids provides a better contrast in neutron scattering to resolve the position of unlabeled M85 in the labeled bilayer using neutron reflectivity.

To obtain high coverage SLBs, a mixture of POPC and other lipids had to be utilized since the deposition of only POPS or POPA would not have been possible or highly difficult. Due to its neutral charge as well as its cylindrical molecular shape, POPC facilitates the correct deposition of SLBs. Therefore, for neutron reflectivity measurements, the following mixtures were adopted: POPC, POPC:POPS ratio 8:2 (mol/mol), Dpol, and POPC:POPA ratio 8:2 (mol/mol), with a final amount of negative charge of 0, 20, 25, and 40% (negative charges over number of lipids). Finally, LUVs used for ThT aggregation assays as well as for TEM measurements were obtained both for these mixtures as well as for the pure POPC, POPS, and POPA since vesicle formation does not require a minimum amount of POPC.

### M85 Variant Is Prone to Fragmentation

**Figure 2:**
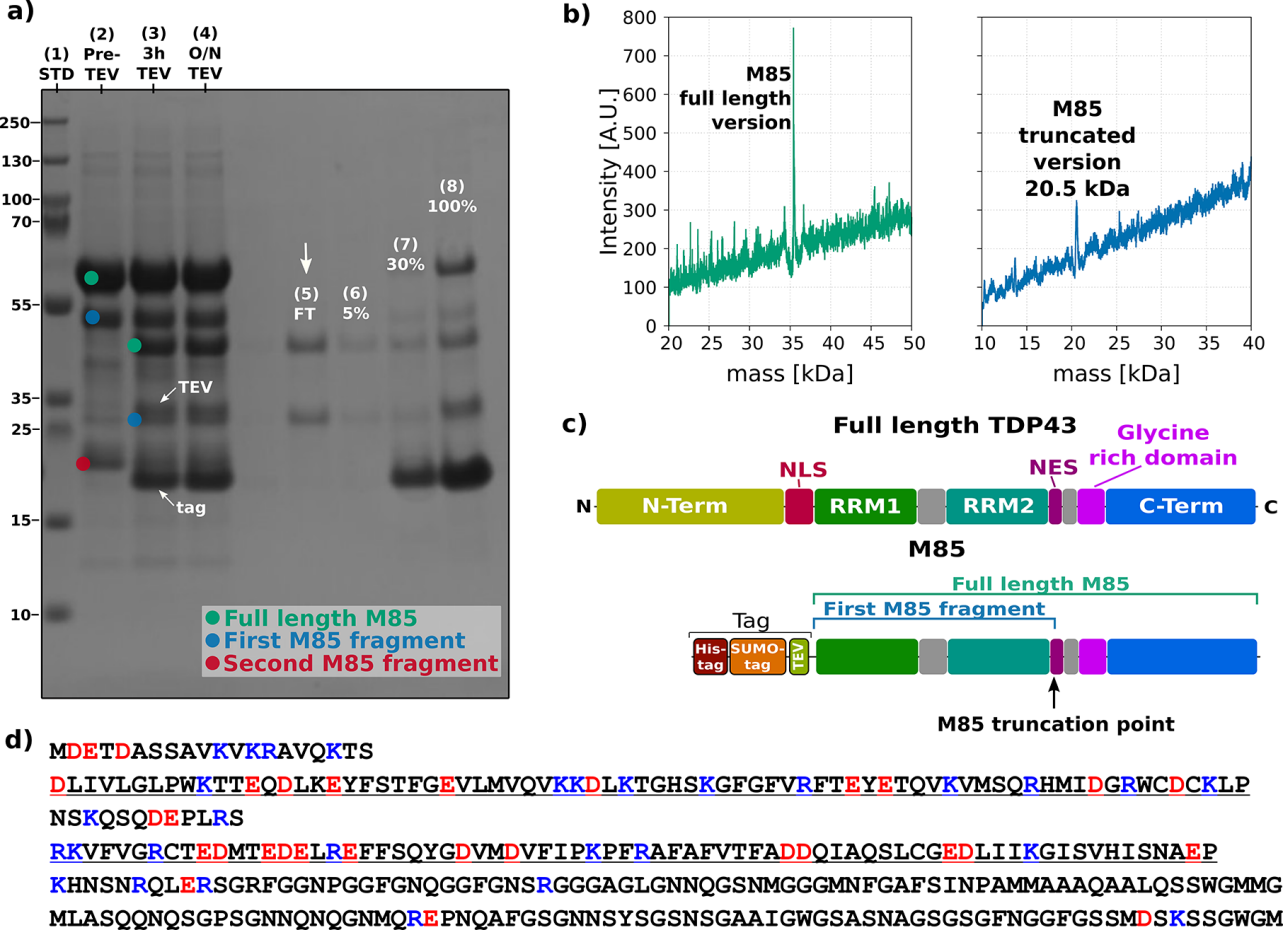
Purification and characterization of the M85 variant. (a) SDS-PAGE of the purification steps of the M85 variant, where lane 1 is the standard, lane 2, 3, and 4 are the sample immediately before addition of the TEV, after 3h, and after overnight incubation, respectively, and lane 5, 6, 7, and 8 are different aliquots during the reversed Ni affinity chromatography. The purified protein (M85 and its fragment) is in lane 5, and they were finally passed through an ion exchange chromatography column to remove contaminants. FT stands for flowthrough, whereas the percentages reported are the percentage of elution buffer utilized to elute that aliquot. (b) Electrospray mass spectrometry analysis of the two M85 fragments separated by using a size exclusion chromatography (SEC). (c) Schematic domain architecture of full-length TDP-43 (top) and the tagged M85 fragment (bottom) where it is highlighted the truncation point. (d) Sequence of the M85 fragment (residues 85–414). The first row contains the residues preceding RRM1 (85–104) expected to be unstructured. RRM1 (105–178) and RRM2 (191–262) are underlined in rows 2 and 4, separated by the linker between the two domains. The region 263–414 contains the unstructured gly-rich region and C-terminus. The RRM domains are defined according to the UniProt annotation. The positively and negatively charged residues are marked in blue and red, respectively. The DNA and amino acid sequences of full-length TDP-43 and of the construct used in this study are provided in the Supporting Information.

TDP-43 is known for its tendency to aggregate and fragment.^35^ Consistent with this knowledge, we observed that M85 also possesses similar behavior. Our observations are based on at least 10 independent purifications of the protein. After optimization, protein expression was always performed at 18 °C overnight to maximize the protein yield while minimizing aggregation (Figure S2a). No fragmentation products were visible before purification, but fragments began to appear after sonication and centrifugation just before the first Ni affinity chromatography step (Figure S2b). After the first Ni affinity and cleavage reaction, the solution was subjected to reverse Ni affinity chromatography to remove the tag and uncleaved protein (Figure 2a). The final samples were passed through an anionic exchange chromatography column to remove all remaining contaminants, concentrated, and stored at −80 °C. The chromatograms of all of the purification steps are provided in Figure S3. A Western blot of the final protein preparation was performed using both anti-TDP-43 and anti-His monoclonal antibodies, confirming that the purified fragment is TDP-43-positive and that the tag was successfully removed (Figure S4). Mass spectrometry (Figure 2b) and SDS-PAGEs indicated that the final protein preparation contained two proteins of 35.5 and 20.5 kDa.

We noticed that, on average, fragmentation happened mostly at the beginning of the purification scheme and that the cleaved samples remained stable over time after the initial events, suggesting a spontaneous rather than an enzymatic mechanism. Attempts to purify the two proteins individually failed due to high amounts of precipitation after concentrating the diluted samples. We concluded that the two fragments need to be copresent at a given ratio to remain soluble, and this mixture was utilized for all the experiments presented in this work.

The 35 kDa band was identified by mass spectrometry as the expected M85 fragment (residues 85–414), while the smaller band was assigned to fragment M85-K263. This fragment is truncated almost exactly at the end of RRM2 and lacks the unstructured C-terminal domain that is thus amenable to cleavage (Figure 2c). This observation agrees with the intrinsically disordered nature of the C-terminus of the protein and further supported by a recent work of the Lashuel group that shows that this region is just outside the core of the fibrils and highly flexible.^49^ We provide a novel hypothesis, however, that fragmentation, likely not caused by enzymatic processing, could be due to the necessity to reach a ratio of protein fragments that optimally increases solubility. It is also worth mentioning that M85 is overall neutral with an isoelectric point (IP) of 6.91, whereas M85-K263 is mildly acidic with an IP of 5.69. This value is almost the same as that of full-length TDP-43 (5.85). However, the charge distribution is such that, locally, the RRM1 and 2 are strongly charged with 28 negatively charged groups versus 24 positively charged residues (excluding histidine residues that, under the conditions used, would be deprotonated) (Figure 2d). The C-terminal domain (residues 264–414) contains instead only a few charges (6 positive and 3 negative charges) and a composition rich in serine, glycine, and asparagine residues that, together, account for 73% of this region.

Together, these data confirm a strong intrinsic tendency of TDP-43 and its fragments to degrade into smaller fragments and suggest a new model to explain TDP-43 spontaneous fragmentation.

### Effect of Lipids on the Protein: Vesicles with Different Lipid Compositions Have Distinct Effects on M85 Aggregation

**Figure 3:**
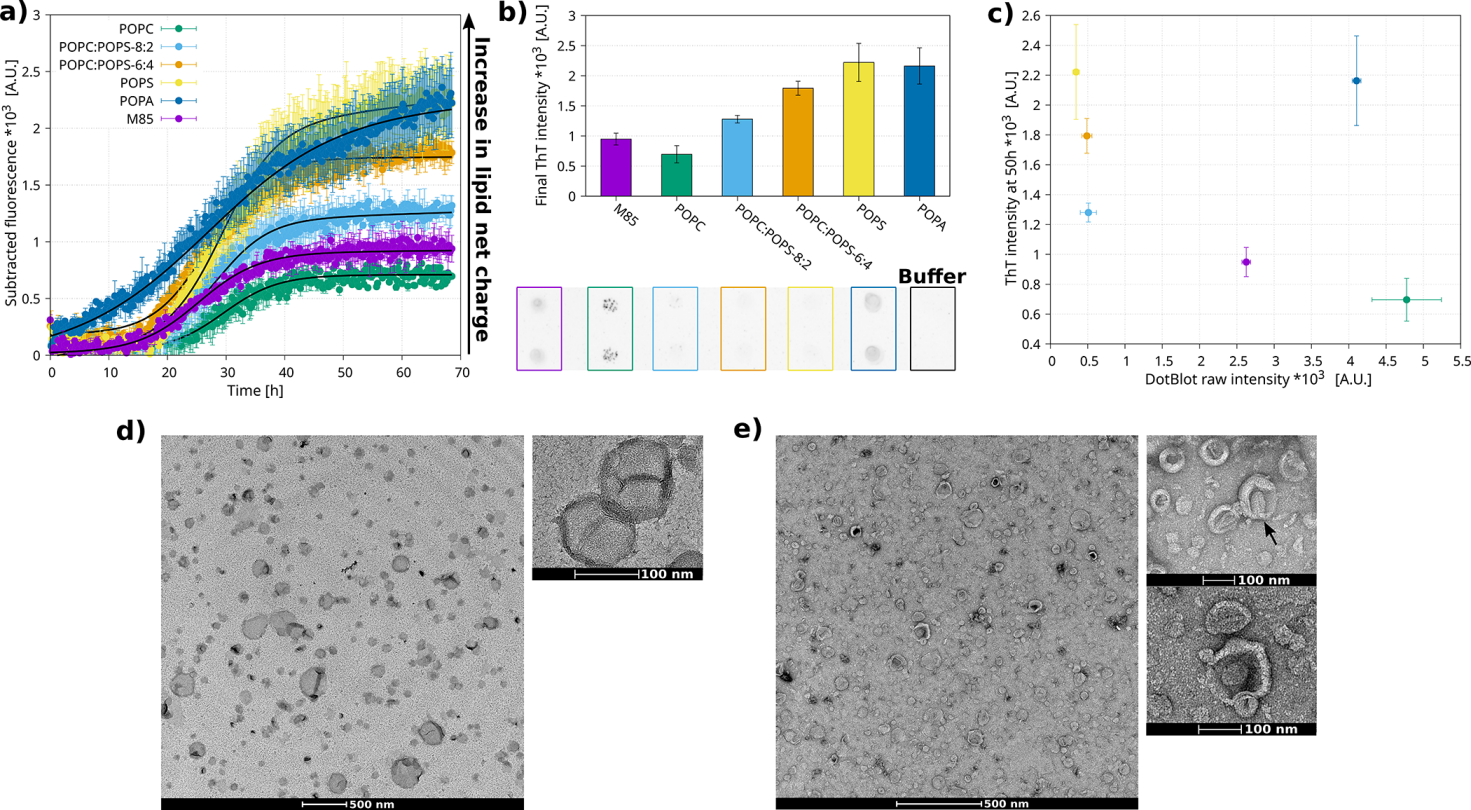
M85 aggregation assay in the presence of lipids. (a) ThT aggregation assay measurements of M85 aggregation kinetics with the presence of different composition LUVs (ThT intensity shown is subtracted by the buffer constituted of only LUVs samples), analyzed using a simple sigmoidal equation (provided in the Supporting Information) to obtain the t50 (displayed in Figure S5b). (b) Final ThT fluorescence intensity of each sample as well as a dot-blot of the supernatant after the aggregation, where duplicates are shown for each sample. (c) Plot of dot-blot intensity versus ThT final intensity. (d, e) Negative stain transmission electron microscopy of POPC:POPS ratio 8:2 (mol/mol) LUVs incubated in the absence (panel d) or in the presence (panel e) of M85 for 4 days. A TEM picture of M85 only is shown in the Supporting Information.

Lipid–protein interactions have been reported to play an important role in mediating the aggregation of several amyloidogenic proteins.^50^ To investigate whether the M85/M85-K263 mixture interacts with lipid membranes, we monitored the aggregation of the protein in the presence of LUVs using the amyloid-sensitive probe ThT.^51^ ThT is a molecular rotor constituted of benzothiazole and benzamine rings that, when inserted into the aggregate structure (within the beta-sheets), the two rings form a planar structure enhancing its fluorescence. Therefore, the amount of fluorescence is proportional to the number of aggregates. By adjusting the lipid composition of the LUVs, we aimed to examine the influence of lipid surface charges on M85/M85-K263 aggregation. LUVs constituted of POPC, POPS, and POPA were prepared as well as the mixtures POPC:POPS ratios 8:2 and 6:4 to modulate the net surface charge (Figure 3). For this experiment, LUV suspensions were used at a final concentration of 100 μM where the M85 was approximately 2 μM in its final concentration.

M85/M85-K263 aggregated after approximately 40 h in the absence of vesicles. The presence of vesicles did not significantly alter the aggregation lag phase of the mixture, except when pure POPA was present, which accelerated the process (no lag phase was indeed observed) (Figure 3a and Figure S5a). Additionally, the ThT intensity at the plateau increased with the negative charge of the lipids, reaching its highest level in the presence of pure POPS or POPA (Figure 3a,b). The final ThT intensity was similar in the presence of pure POPS and POPA, suggesting a comparable conversion of monomers into aggregates in the presence of these two lipids. In contrast, the aggregation kinetics of M85/M85-K263 in the presence of pure POPC showed a plateau that was lower with respect to the control.

This result suggests that some monomers were sequestered by the neutral membrane, reducing the protein concentration and thus its availability for aggregation. Altogether, these results indicate that an increase in negative charge on the lipid membranes leads to an acceleration of the aggregation process and an increase in the final yield of aggregates (Figure 3a,b).

To confirm that the higher ThT intensity at the plateau corresponds to a greater aggregate yield, we quantified the residual soluble protein at the end point of aggregation under the various lipid conditions. Samples were centrifuged to separate the soluble proteins from the lipid fraction. The supernatant, containing the soluble proteins, was analyzed using dot-blot to assess protein content (Figure 3b). The spots were integrated, and the raw data were plotted against the final ThT fluorescence (Figure 3c). An inverse correlation was observed between the final ThT fluorescence values and levels of soluble M85 (Figure 3c), indicating that the ThT fluorescence reliably reflects the aggregate yield (except in the case of POPA). This implies that all nonsoluble protein is in the ThT-positive aggregates. This behavior is similar to that observed by Galvagnion et al. in the case of lipid-mediated α-synuclein aggregation,^39^ suggesting that M85 may be able to aggregate via heterogeneous primary nucleation^52^ when exposed to lipid membranes. Interestingly, the sample containing POPA deviated from this trend, showing an unexpectedly large amount of soluble protein relative to its ThT intensity. We interpreted these results as the presence of ThT-positive aggregates that are too small to be separated by the centrifugation conditions used here (14,000*g* for 1 h).

### Effect of the Protein on the Lipids: M85 Interacts with and Affects the Stability of the Bilayer

Previous studies have suggested that TDP-43 is able to interact with biological membranes^38^ but the interaction has not yet been characterized. To investigate the mechanism of interaction, we carried out measurements using microscopy, as well as biophysical techniques. We performed transmission electron microscopy (TEM) measurements on LUVs with a POPC:POPS molar ratio of 8:2 in the absence and presence of M85 to investigate how the protein affects the structure of LUVs (Figure 3d,e). We selected this lipid composition because it provides optimal uranyl acetate staining for TEM. We found that the morphology of LUVs greatly differed under the two conditions. In the absence of protein, LUVs appear round and structurally intact (Figure 3d). In the presence of the protein, LUVs appeared to collapse (such as the vesicle highlighted with an arrow in Figure 3e), suggesting pore formation and structural damage of the vesicles. Additionally, debris were visible in the protein-containing condition but not in its absence, indicating that M85/M85-K263 mixture can compromise the structural integrity of LUVs disrupting the bilayer structure according to a mechanism of membrane disruption suggested by the Ulmschneider team.^38^ These debris could be associated with complexes of protein and lipids, as well as protein aggregates. A TEM image of the only M85 sample is available in Figure S6.

To investigate the interaction between M85/M85-K263 and lipid membranes, we preliminarily used ellipsometry measurements, a technique that, although working on lipid monolayers, can rapidly provide information about the interface structure before carrying out more complex experiments using a neutron source (Figure S7). These measurements detect a change in polarization as light reflects from an interface. The measured response depends on the optical properties and thickness of the specific material at the interface. In this experiment, the M85/M85-K263 sample was injected into the bulk solution beneath a lipid monolayer containing pure neutral POPC. After approximately 1 h, ellipticity began to increase, leading to higher surface excess due to protein insertion, which reached a plateau after approximately 6 h. Although this technique cannot separate the contributions of the signals of protein and lipids because of the similar refractive indices and the limited resolution of single-angle kinetics measurements, these results strongly suggest that the M85/M85-K263 mixture interacts with the lipid monolayer, penetrating it over time.

### Lipid Charge Influences How M85 Interacts with the Lipid Bilayer

Given the fragmentation propensity of M85, we first assessed whether both the M85 and M85-K263 fragments interact with the membranes. To do so, we incubated the M85/M95-K263 mixture with LUVs constituted of different lipid compositions. Two aliquots were taken: time zero (as soon as LUVs and M85 mixture were mixed) and after an overnight incubation. The two aliquots were then centrifuged to separate the LUVs from the soluble fraction. Next, the quantification of the presence of the M85 fragments in the LUVs fraction was carried out using a Western blot (anti-TDP-43 antibody) (Figure S8a,b). We found that the species interacting with membranes are predominantly M85 at time zero. However, after overnight incubation, approximately equal amounts of M85 and its fragment are present in the membrane fraction. Since both fragments can bind to membranes to the same extent, we speculate that the binding domain is present in both proteins. The rapid membrane binding of M85, compared to the M85-K263 fragment, suggests that the C-terminal domain (residues 264–414) influences the binding kinetics but not their affinity. Overall, these experiments indicate that although the M85 sample contains a mixture of two proteins, membrane binding mainly occurs through the RRM region (RRM1 and RRM2 domains, residues 105–178), which is shared by both fragments.

**Figure 4:**
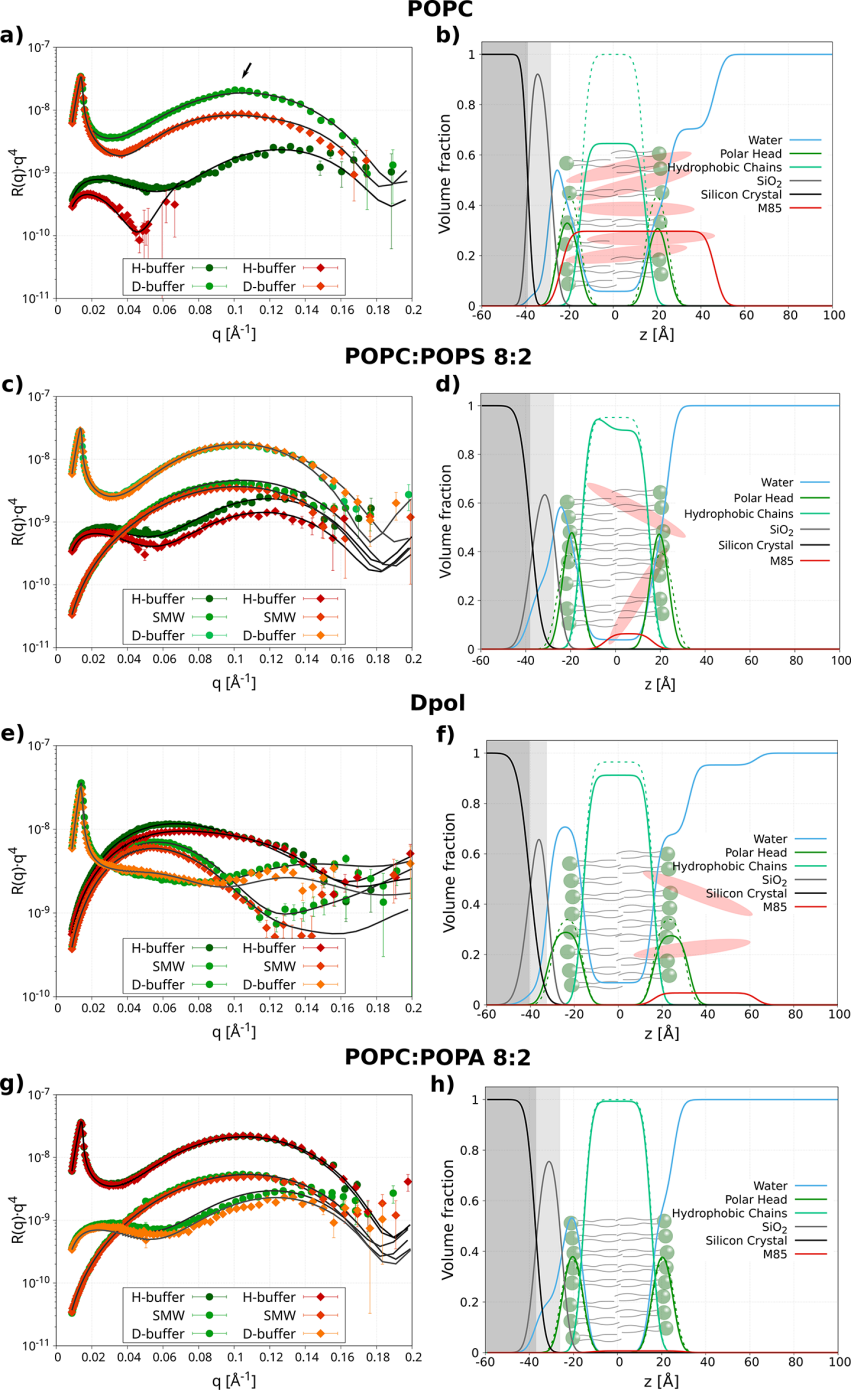
Fitting and analyses of neutron reflectivity data before (green colors) and after (red colors) the M85 addition under different SLB compositions. (a, b) POPC SLB, highlighted with an arrow is a broad Bragg peak most probably due to diluted multilamellar formations caused by issues in the vesicles fusion process, (c, d) POPC:POPS 8:2, (e, f) Dpol and (g, h) POPC:POPA 8:2 SLB. For the panels on the left-hand side (a, c, e, and g) in green color and in red color are data before and after ca. 20 h from the M85 injection, respectively. The solid lines represent the minimized theoretical curves. On the right-hand side (b, d, f, and h), the minimized models, where the dashed lines and solid lines represent the model before and after M85 injection, respectively. M85 protein is represented in red color.

To further characterize the interaction between M85/M85-K263 and membranes at the molecular level, we performed neutron reflectivity measurements on the SLBs (Figure 4). This technique has angstrom resolution along the *z*-axis of the sample (*i.e.*, the axis perpendicular to the plane of the SLB), enabling identification of the position of the protein along the structure of the membrane as well as the structural damage of the lipid membranes caused by the interaction. Reflectivity measurements were performed using SLBs with the following lipid compositions: POPC, POPC:POPS, and POPC:POPA mixtures at molar ratios 8:2, and a polar lipid extract from the *Pichia pastoris* yeast.^41^ The measurements were performed as follows: SLBs were characterized in two or three buffer contrasts (H-buffer, D-buffer, and SMW): this is a standard method in neutron reflectivity since the scattering length (approximately equivalent to a refractive index for neutrons) is enormously different between H and D atoms, allowing the same samples to be measured with different contrasts, increasing the accuracy of the fitted parameters. Next, the M85/M85-K263 solution (nominal concentration 2 μM) was injected into the neutron reflectivity cell, and kinetics measurements were carried out. An incubation of ca. 20 h was carried out after which the neutron reflectivity cell was washed with H-buffer, and the SLB was measured again in two or three contrasts. The data analysis shown here was performed in the SLBs before the M85 mixture injection and after the incubation and cell wash. In the data analysis, the protein component was approximate to a cylinder with a fixed volume, and a height (thickness) was obtained by dividing the volume by its area.

Having a zero net charge, the POPC membrane can be utilized to assess the interaction of the M85/M85-K263 mixture with a neutral zwitterionic lipid membrane (Figure 4a,b). Upon injection of the M85 mixture, the protein was incorporated into the lipid membrane, positioning between −24 and 46 Å (Table 1), where the 0 value is the center of the SLB, *i.e.*, between the two leaflets. Therefore, M85 proteins were found to be placed inside the SLB, protruding also toward the bulk solution (Figure 4b). The amount of M85 incorporated after ca. 20 h was found to be around 24 ± 4 (lipids/M85 mol/mol) (Table 1), leading to a fraction volume of 0.3 (30% in volume of M85 in the SLB) (Table 2). The insertion caused the topological area per lipid to a decrease of ∼3 Å^2^ probably due to the compression of POPC molecules in the SLB in response to the insertion of the protein. Interestingly, the interaction of M85 with the POPC SLB was found to displace POPC lipids from the SLB to the bulk solution. We found that around 38% of the initial amount of phospholipid molecules in the SLBs were removed from the membrane, hypothetically incorporated within the aggregates in the bulk solution. These results agree well with our observation that the neutral POPC has a smaller effect on the aggregation of M85, most probably due to the high sequestration of M85 monomers. Additionally, an increase from 0 to 8% of defects in water was found in the SLBs after M85 insertion, indicating damage of the membrane (Table 1).

**Table 1:** Reflectivity Parameters of the Four Samples Utilized before and after the Addition of M85^a^

SLB samples	POPC bilayer		POPC:POPS bilayer		Dpol *Pichia pastoris* bilayer		POPC:POPA bilayer	
NR parameters	–M85	+M85	–M85	+M85	–M85	+M85	–M85	+M85
*t*_PH_ [Å]	11 ± 1	10 ± 1	11 ± 1	8 ± 1	15 ± 1	16 ± 1	10 ± 1	10 ± 1
*t*_HC_ [Å]	15 ± 1	15 ± 1	15 ± 1	15 ± 1	16 ± 1	16 ± 1	16 ± 1	15 ± 1
*A*_lipid_ [Å^2^]	63 ± 2	60 ± 2	62 ± 2	60 ± 1	59 ± 2	59 ± 2	59 ± 2	60 ± 2
ϕ_PH_ [%]	53 ± 2	55 ± 8	61 ± 1	45 ± 3	68 ± 1	77 ± 1	59 ± 1	58 ± 1
ϕ_defects_ [%]	0 ± 0	8 ± 2	5 ± 1	4 ± 1	4.0 ± 0.2	8.8 ± 0.3	0 ± 0	0 ± 0
*z*_M85_ [Å] (min;max)		–24; 46 ± 2		–5; 17 ± 3		17; 63 ± 3		not relevant
ψ_M85_ (mol_lipids_/mol_M85_)		24 ± 4		489 ± 25		305 ± 38		not relevant

a *t*_PH_ and *t*_HC_ are the thicknesses of the polar head and chain region, respectively; *A*_lipid_ is the topological area per lipid; ϕ_PH_ is the amount of water in the polar heads; ϕ_defects_ is the fraction volumes of water through the SLB (defects); zM85 is the position in Å of the M85 fraction volume distribution; and ψ_M85_ is the ratio between lipid and M85 molecules in or on the SLB (expressed in mol/mol).

**Table 2:** Parameters Derived from the Reflectivity Analysis^a^

SLB samples	ϕ_HC_	ϕ_defects_	ϕ_M85_	lipids removed [%]	bilayer charge [%]
**POPC**	0.62	0.08	0.30	38	0
**POPC:POPS 8:2**	0.90	0.04	0.06	5	20
**Dpol**	0.91	0.09	0.05^b^	5	≈25
**POPC:POPA 8:2**	1.00	0.00	0.00	≈0	40

a The displayed parameters refer to the center of the SLB (z = 0). ϕ_HC_ is the fraction volume of the hydrophobic chains; ϕ_M85_ is the fraction volume of M85 in the SLB; and ϕ_defects_ is the amount of buffer in the SLB (damage of the SLB).

b Fraction volume of the M85 outside the SLB (not at *z* = 0).

In the case of POPC:POPS (8:2) SLBs (Figure 4c,d), the 20% increase of negative charge in the lipid membrane as compared to pure POPC SLBs was found to have a drastic effect on the interaction since lower amounts of protein were incorporated in the membrane (ca. 500 lipids/M85 mol/mol leading to a volume fraction of 0.06 (6%) of M85 in the SLBs (Tables 1 and 2). Additionally, a shift in the position of the proteins along the *z*-axis was found toward the bulk solution (more in the external leaflet) as compared to the POPC membrane (Figure 4c,d). In fact, the average position of M85 was found to be between −5 and 17 Å, compared to POPC that was between −24 and 46 Å. Also in this case, the topological area per lipid was found to decrease probably due to the same lipid compression that we previously discussed for the POPC SLB. In this case, a small amount of lipids was found to be removed from the SLBs (around 5%), where the lipids removed were substituted by protein insertion (Table 2). In fact, in this case, the amount of defect water in the SLBs was not found to vary significantly.

In the presence of a lipid bilayer composed of deuterated natural phospholipid extracts (Figure 4e,f), the insertion of the protein was found to be more superficial as compared to 8:2 POPC:POPS and POPC samples (pushing the average M85 position to values between 17 and 63 Å; Tables 1 and 2). This behavior could be caused by the higher charge of these membranes (around 25% as compared to pure POPS) and the presence of additional phospholipid classes (such as PE, PG, and CL apart from PC and PS). Therefore, it appeared that a higher charge of the lipids leads to a more superficial interaction of M85 with lipid membranes. Interestingly, the amount of lipids removed (around 5%) is similar to that observed with the POPC:POPS 8:2 sample. The fraction volume of M85 found interacting with this SLB was 0.05 (5%) with a ratio lipids to M85 of around 300 (mol/mol). In this case, the area per lipid did not change most probably due to the superficial interaction of M85/M85-K263 with this SLB.

Interestingly, when M85/M85-K253 was exposed to the more negative POPC:POPA (8:2) SLBs, no interaction was observed (Figure 4g,h and Tables 1 and 2). Only slight differences of the membrane structure were observed between measurements performed before and after the protein injection, likely caused by a slight change in hydration of the membrane. This might be the consequence of the interaction being so transient or superficial that the protein is washed away by the phosphate buffer prior to the final measurement. No lipids were found to be removed in this sample. All of the SLD profiles from this analysis are available in Figure S9.

Altogether, the neutron reflectivity data suggest that a higher negative charge on SLBs promotes an interaction of M85/M85-K263 that is primarily localized on the membrane surface rather than penetrating the hydrophobic core.

These results provide a possible explanation for the aggregation behavior observed in the ThT fluorescence experiments. When the SLB contained pure POPC, the M85/M85-K263 monomers bind to the membrane hydrophobic core tightly. The monomers would be sequestered from the bulk of the solution and cannot aggregate. In the presence of negatively charged SLBs, M85/M85-K263 monomers transiently interact with the surface of the membrane, which acts as a nucleation site for the aggregation of the protein.

### Kinetics of the Interaction Recorded Using Neutron Reflectivity

In the previous section, we showed that higher lipid charges lead to more superficial interactions, reducing the level of protein binding to the SLBs (Figure 4). ThT data also indicated that higher charges increase the number of aggregates (Figure 3). Thus, we speculated that higher charges may cause the M85/M85-K263 mixture to interact more superficially and transiently with lipid membranes, leaving more protein available to aggregate. To confirm this hypothesis, we analyzed the neutron reflectivity measurements collected at different time points after protein injection to extract the kinetics of the interaction.

**Figure 5:**
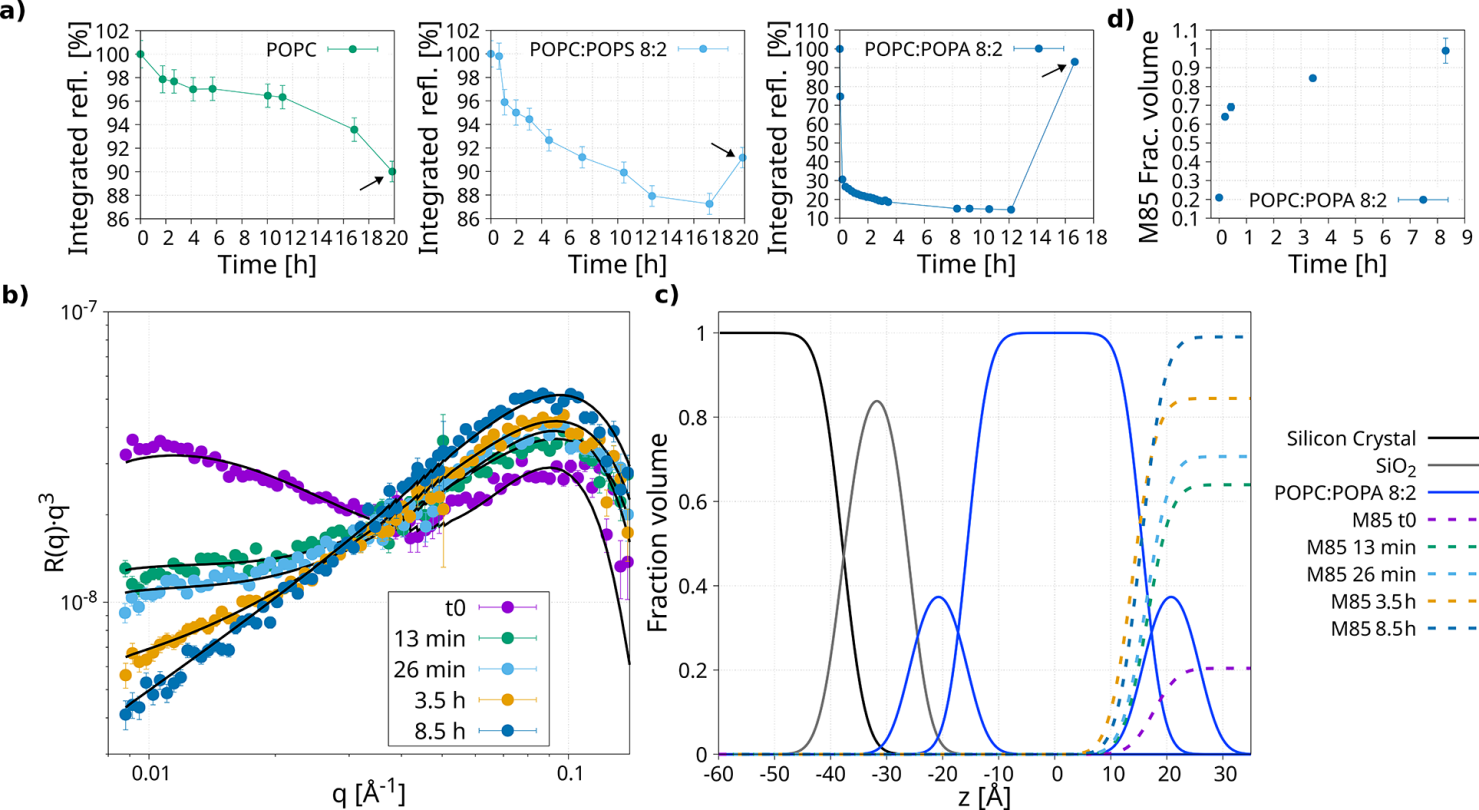
Kinetics of interaction. (a) Trend of the neutron reflectivity-integrated kinetics measurements for the samples POPC, POPC:POPS 8:2, and POPC:POPA 8:2, highlighted with the arrows is the data set after the washing of the cell with buffer (analyzed and discussed in Figure 4). (b) Kinetics reflectivity profiles and analysis of the sample containing M85 and POPC:POPA 8:2 measured in H-buffer. (c) Minimized model from the analysis of the data in panel (b), where the fraction volume distribution of the deposition layer is shown with dashed lines. The “t0” is the data set collected just after the M85 injection into the cell (∼4 min from the injection). (d) Plot of the fraction volume of the deposition layer versus time.

Time-course kinetic measurements were carried out for the samples of POPC:POPA 8:2, POPC:POPS 8:2, and POPC. Figure 5a shows the integration of the neutron reflectivity kinetic profiles over time, where it can be noticed how an increase in charge accelerates the interaction between the protein and lipid membranes, as indicated by the faster reduction in the integrated reflected intensity with more highly charged SLBs. Interestingly, for the sample POPC:POPA 8:2 in which the M85/M85-K263 mixture was not detected after washing the cell, there was a strong and high-rate interaction happening within the first 2 h after injection (Figure 5a). However, after the washing step, the signal returned to the value before injection (point highlighted with the arrow). A detailed analysis (Figure 5b,c) of the time-course was performed on this sample. The reflectivity profiles were fitted (Figure 5b), and the final minimized model is shown in Figure 5c.

The interaction observed at these lipid ratios could not be modeled assuming the binding of monomeric M85/M85-K263. In fact, the thickness of the component representing the M85 deposition onto the SLBs (“deposition layer”) was found to be larger than what neutron reflectivity can observe. Therefore, this “deposition layer” was modeled as a component with an undefined thickness (Figure 5c, dashed lines). The fact that the layer was found to be this thick implies aggregate formation, while the M85/M85-K263 interacts with the SLBs. This is in agreement with our ThT data, where the lag phase is clearly absent in the POPA sample, leading to protein aggregation, even during the early stages of the protein–lipid interaction. After the protein injection, the deposition layer grows and the fraction volume increases over time until it reaches the value of 1, fully covering the SLB surface. A plot of the fraction volume parameter change over incubation time is provided in Figure 5d. This deposition layer was found to be only located superficially on the SLBs, and the fact that it is removed when the neutron reflectivity cell was washed before the final measurements suggests this as a transient interaction.

In the presence of the SLBs composed of POPC:POPS 8:2, M85/M85-K263 was found to first deposit onto the membrane surface, suggesting an interaction with the phospholipid head groups, similar to what was observed for POPC:POPA 8:2. Insertion of the protein into the membrane was observed after approximately 6 h (Figure S10). The protein interacting with the surface of the SLBs was washed away by the washing step of the cell, while the inserted portion remained embedded in the SLBs. This process can also be observed by the signal increase in the integrated reflectivity profile (Figure 5a, point highlighted with the arrow), similarly to what was observed for POPC:POPA 8:2.

In contrast, in the presence of POPC, the protein started immediately to be embedded into the lipid membrane (without the superficial deposition), and the washing step did not remove a significant amount of protein from the membrane, suggesting that the protein is stably embedded within the lipid bilayer (kinetic data not shown). However, since the higher level of noise in the measurement prevented a complete analysis and introduced significant errors in the minimized parameters, this result should be further confirmed.

## Discussion

Much interest has been concentrated in the last 5–10 years on understanding the role of electrostatics in the liquid–liquid phase separation of TDP-43 and the formation of membraneless organelles, which is an important aspect of TDP-43 cellular function. Our work investigated a different aspect: the interaction between TDP-43 and phospholipids. This focus was motivated by the substantial evidence supporting vesicle-mediated spreading of TDP-43 aggregates and by the complete lack of biophysical characterization of the interactions between TDP-43 and phospholipids. Ours is, to our knowledge, the first comprehensive study that attempts to understand the atomic determinants and molecular consequences of the interaction and that uses a strongly aggregation-prone TDP-43 fragment. Our results highlight the importance of the relative charges of both the protein and lipids^53^ and thus indicate an interplay between electrostatic and hydrophobic forces acting in this interaction.

EVs have been originally classified as lipid particles released from reticulocytes to get rid of unwanted material, including proteins, lipids, and nucleic acids. The perspective has now significantly changed, and EVs are now considered important means for intercellular communication,^54,55^ immune system activation,^18,56^ cellular homeostasis,^57^ environmental adaptation,^15^ and cell maturation and migration.^58,59^ It is also now thought that EVs can contribute both to physiological and pathological processes in the central nervous system, although little is known about the details of this dual role.^60−62^

We selected the M85 fragment since protein fragmentation appears to be a common posttranslational modification in neurodegeneration, particularly TDP-43 pathologies. Furthermore, a 35 kDa fragment was recently described to be associated with EVs.^33,34^ We found that M85 further fragments to produce a shorter version covering the region M85 to K263. This is interesting because this region contains the two RRMs (residues 105–178) that represent the RNA-binding region of the protein. RRMs are believed to be primary functional elements of the protein and are known to play a key role in RNA metabolism. We attempted to further purify the two fragments, finding that the process would strongly affect the solubility and lead to the precipitation of the individual species. This evidence suggests that protein fragmentation could be dictated by the optimization of fragment mixtures that permit protein solubility and retention of functionality. A similar concept might be important for the mixture Aβ40/Aβ42 in which the less soluble Aβ42 coexists in the brain under normal physiological conditions in an Aβ42:Aβ40 ratio of ∼1:9.^63,64^ This ratio is shifted to a higher percentage of Aβ42 in brains of patients with familial AD, and this has been shown to lead to increased synaptotoxicity.

We found that the M85 and M85-K263 fragments appear to bind to membranes to a comparable extent but with different kinetics, indicating that membrane binding mainly occurs through the RRMs region, which is shared by both fragments. This observation does not come as a complete surprise if we want to explain the role of electrostatics in the interaction giving that most of the charges cluster in the RRM domains but also underlines once again the duality between physiologic function and aberrant aggregation, already observed in other proteins involved in neurodegeneration.^65^ On the other hand, the C-terminal domain seems to play a kinetic role in the interaction. In fact, our results indicate that the M85-K283 fragment requires a longer incubation time to bind the membranes. It is well-established that the C-terminal domain is involved in TDP-43 aggregation. We may speculate that the overall hydrophobicity of the C-terminal domain may accelerate lipid interaction by engaging with the core of the lipid bilayer, as suggested by simulations of the full-length TDP-43.^38^

Our findings also reveal that the M85/M85-K263 mixture interacts with lipid membranes in distinct ways based on the charge characteristics of the phospholipids. We tested both *in vitro* assembled mono- and bilayers containing phospholipids commonly found in eukaryotic membranes, and, for comparison, membranes obtained from yeast extracts which, although still distant from neuronal membranes, have compositions that can more faithfully mimic the *in vivo* environment and set the scenario for further studies. Although RRM1 and RRM2 bind to RNA, this behavior is reasonable because they have a negative net charge. We observed that M85/M85-K263 inserts into the lipid bilayer of zwitterionic membranes. This mechanism would have the obvious effect of reducing the overall concentration of the protein in solution, which is available to aggregate. As the membrane negative charge increases, the protein moves to the bilayer’s phospholipid surface. This could allow the membrane to serve as a nucleation site for monomeric M85/M85-K263 to aggregate. The protein interaction with lipid membranes would also disrupt the structural integrity of the lipid bilayer. The insertion within the core of the membrane causes the release of phospholipids into the solution, ultimately leading to the collapse of the membrane. Our experimental data are also supported by computer simulations, which show that a peptide encompassing the Gly-rich region of the TDP-43 C-terminal domain can theoretically form pores within a 1,2-dimyristoyl-*sn*-glycero-3-phosphocholine (DMPC) bilayer.^38^ We speculate that membrane pore formation represents a potentially pathological mechanism, as it has been found for other amyloidogenic proteins, such as α-synuclein^66^ and amyloid-β^67^ contributing to membrane damage and cellular dysfunction.

Next, we considered the significance of our results for neurons and how we could extend our findings to full-length TDP-43. Neurons are known to contain a high concentration of cholesterol (>20%), which should anyway contribute to a predominantly neutral membrane charge. Although we have by choice neglected cholesterol in this study to focus on the role of charges, this suggests that the *in vivo* environment may more closely resemble conditions modeled with neutral or mildly negative lipids. However, the lipid composition of EVs can be significantly different from that of neuronal membranes. EVs are typically more negatively charged, whereas neuronal membranes maintain a more neutral charge.^68^ This difference in charge across biological membranes within the central nervous system motivated our systematic studies, which explored the effects of varying membrane charges. Full-length TDP-43 could be thought to behave like the M85-K263, given the similar IP of the two proteins, although it is even more insoluble than its fragments, impacting its purification and experimental handling. A further level of complexity arises when considering the role of posttranslational modifications in the interaction with membranes since TDP-43 is known to be heavily modified according to the pathway involved. This, however, might only be possible when a clearer picture of the modifications becomes available.

## Conclusions

Altogether, our findings about how M85 interacts with lipid membranes based on their charge help us to better understand its tendency to form aggregates and its potential toxic effects. By testing how M85 behaves with different membrane charges, we could see how it might interact with neutrally charged neuronal membranes and negatively charged EVs. These interactions could affect the way TDP-43 and its fragments form aggregates, damage membranes, or move between cells, which are important steps in its harmful role. The next important step would be to test the interactions in the presence of RNA.

## Supporting Information Available

Additional experimental details, materials, and methods, including figures representing the construct sequences, the protein purification and characterization, description of the aggregate kinetics, and additional ellipsometry data (PDF)
