# Supplementary material for: Membrane Charge Drives the Aggregation of TDP-43 Pathological Fragments
Source: J Am Chem Soc. 2025 Apr 8;147(16):13577–91. doi: 10.1021/jacs.5c00594 (PMC12022992; doi:10.1021/jacs.5c00594)
Supplement: Supplementary file 1 — ja5c00594_si_001.pdf [file ja5c00594_si_001.pdf]

Membrane Charge Drives the Aggregation of TDP-43 Pathological Fragments

Giacomo Corucci[1], Devkee M. Vadukul[1], Nicolò Paracini[2,3], Valérie Laux[2], Krishna C. Batchu[4], Francesco A. Aprile[1,5]* and Annalisa Pastore[6,7,8]*

1- Department of Chemistry, Molecular Sciences Research Hub, Imperial College London, London W12 0BZ, U.K.
2- Institut Laue Langevin, Avenue des Martyrs 71, 38000 Grenoble, France;
3- New affiliation: European Spallation Source ERIC, Data Management and Software Centre, Asmussens Allé 305, 2800 Lyngby, Denmark
4- Institut Laue Langevin, Avenue des Martyrs 71, 38000 Grenoble, France
5- Institute of Chemical Biology, Molecular Sciences Research Hub, Imperial College London, London W12 0BZ, U.K.
6- Institute of Brain Sciences, Burlington Danes, The Hammersmith Hospital, Du Cane Road, London W12 0NN, UK
7- The Wohl Institute, King's College London, 5 Cutcombe Rd, London SW59RT, UK
8- Elettra Sincrotrone Trieste, s.s. 14 km 163,500, Area Science Park, 34149 Basovizza Trieste, Italy

Equation S1 – Sigmoidal function utilized for the aggregation profiles analysis

$$S(x) = \frac{1}{1 + e^{-k\cdot(x-x_\mu)}} * S_{int} + S_{min} + m * x$$

Where k is the slope of the sigmoidal function, x_μ is the position of the sigmoidal function in x, S_{int} and S_{min} are the max and minimum y intensity of the sigmoidal function. The last portion of the equation is a straight-line representing aggregation that might happen after the plateau, where m is the slope factor of this straight line.

Figure S1 – Amino acid of M85 within the context of full-length TDP-43 and the plasmid used.

A) Sequence of full length TDP-43 with the M85 sequence in red and the estimated M85 first fragmentation product sequence highlighted in yellow:

MSEYIRVTEDENDEPIEIPSEDDGTVLLSTVTAQFPGACGLRYRNPVSQCMRGVRLVEGILHAPDAGWGNLVYVVNYP
KDNKRKMDETDASSAVKVKRAVQKTSDLIVLGLPWKTTEQDLKEYFSTFGEVLMVQVKKDLKTGHSKGFGFVRFTEYE
TQVKVMSQRHMIDGRWCDCKLPNSKQSQDEPLRSRKVFVGRCTEDMTEDELREFFSQYGDVMDVFIPKPFRAFAFVT
FADDQIAQSLCGEDLIIKGISVHISNAEPKHNSNRQLERSGRFGGNPGGFGNQGGFGNSRGGGAGLGNNQGSNMGG
GMNFGAFSINPAMMAAAQAALQSSWGMMGMLASQQNQSGPSGNNQNQGNMQREPNQAFGSGNNSYSGSNS
GAAIGWGSASNAGSGSGFNGGFGSSMDSKSSGWGM

B) M85 fusion protein sequence expressed from the expression vector with the M85 sequence indicated in red, the TEV recognizing sequence in green and the protein tag in blue:

MGSSHHHHHHGSGLVPRGSASMSDSEVNQEAKPEVKPEVKPETHINLKVSDGSSEIFFKIKKTTPLRRLMEAFAKRQGK
EMDSLRFLYDGIRIQADQTPEDLDMEDNDIIEAHREQIGGENLYFQGMDETDASSAVKVKRAVQKTSDLIVLGLPWKTT
EQDLKEYFSTFGEVLMVQVKKDLKTGHSKGFGFVRFTEYETQVKVMSQRHMIDGRWCDCKLPNSKQSQDEPLRSRKV

FVGRCTEDMTEDELREFFSQYGDVMDVFIPKPFRAFAFVTFADDQIAQSLCGEDLIIKGISVHISNAEPKHNSNRQLERS
GRFGGNPGGFGNQGGFGNSRGGGAGLGNNQGSNMGGGMNFGAFSINPAMMAAAQAALQSSWGMMGMLASQ
QNQSGPSGNNQNQGNMQREPNQAFGSGNNSYSGSNSGAAIGWGSASNAGSGSGFNGGFGSSMDSKSSGWGM

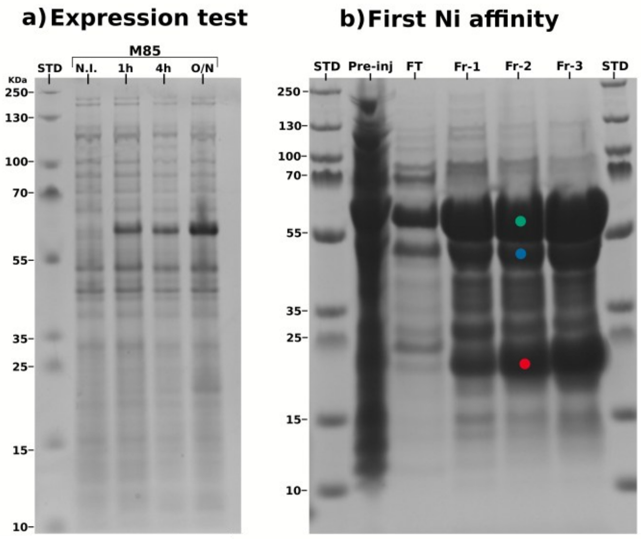

Figure S2 – a) SDS-PAGE gels for expression test, where a single band representing the tagged-M85 appears during its expression. The “N.I.” lane is the non-inducted cell extract and “1h”, “4h” and “O/N” are after one hour, four hours and overnight of induction respectively. b) First Ni affinity aliquots (before and after the purification), where fragments started to appear. The “Pre-inj” lane corresponds to the sample prior the injection into the Ni affinity, “FT” is the flowthrough coming from the Ni affinity column and the “Fr-1”, “Fr-2” and “Fr-3” are fractions collected during the elution step. The standard ladder values are expressed in KDa.

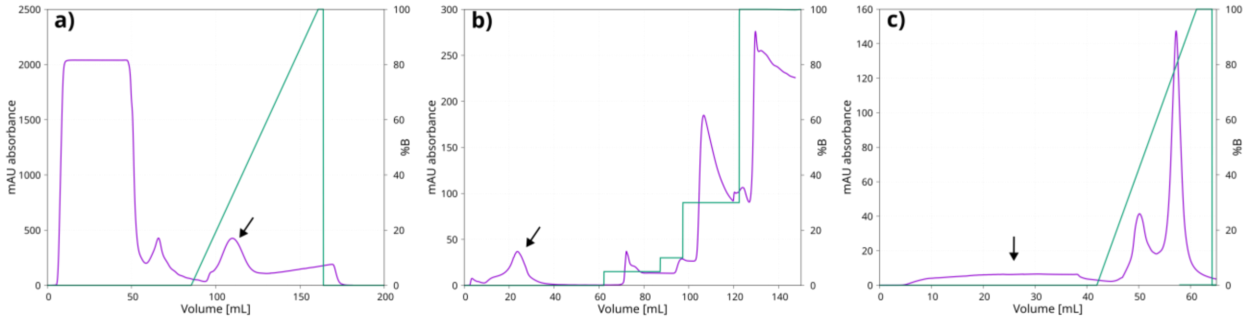

Figure S3 – Chromatograms of the a) first Ni affinity, b) second Ni affinity and c) final IEX to remove any aggregates and contaminants that might be present in the final product. Arrows indicate the position of M85 in the elution profile.

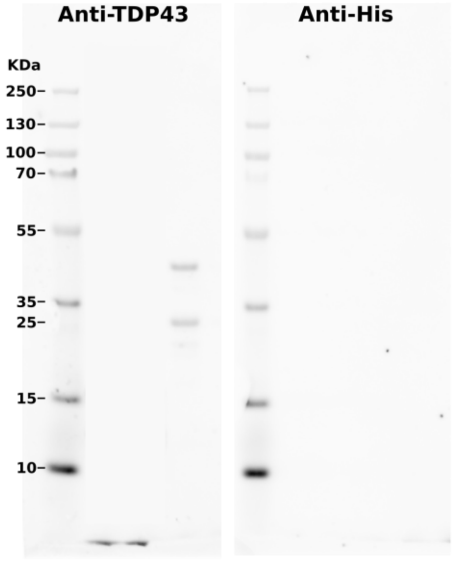

Figure S4 - M85 preparation detected using an anti-TDP-43 and an anti-His antibodies. It can be noticed that two clear bands in the anti-TDP43 WB appear, proving how M85 during the purification process fragments. No visible bands were detected in the anti-His antibody wester blot, showing the free tag M85 prep. The standard ladder values are expressed in KDa.

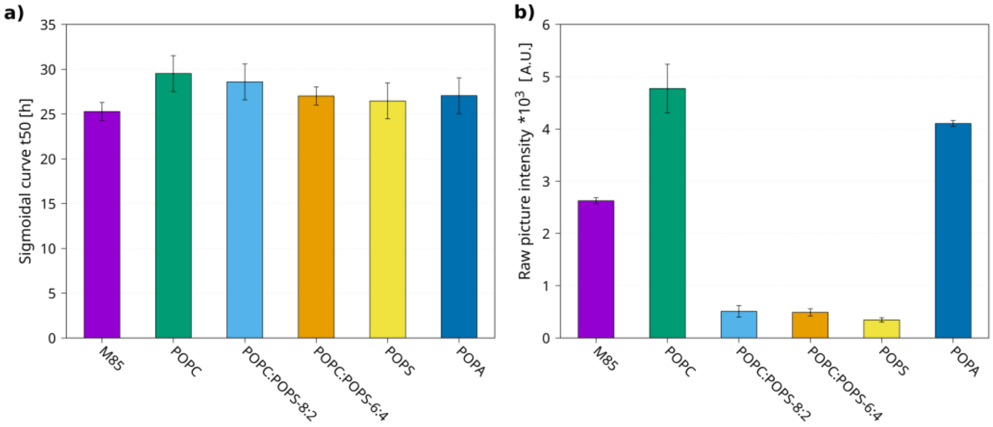

Figure S5 – a) Graph showing the calculated t50 from the aggregation profiles analysis shown in Figure 3a in the main text. The t50 appears to be similar between the different samples, where probably a slight shift of the aggregation profile is present in the POPC compared to the M85 alone. Upon addition of the charge, this value appears to decrease a little until the plateau occurs. b) Graph showing the raw intensities from the dot blot, where POPC seems to decrease the final amount of aggregates in solution whereas lipids with charge increase this amount except for the POPA. In fact we have hypothesized in the main text that this lipid could lead to the formation of smaller aggregates, that can not be separated from the supernatant using centrifugation.

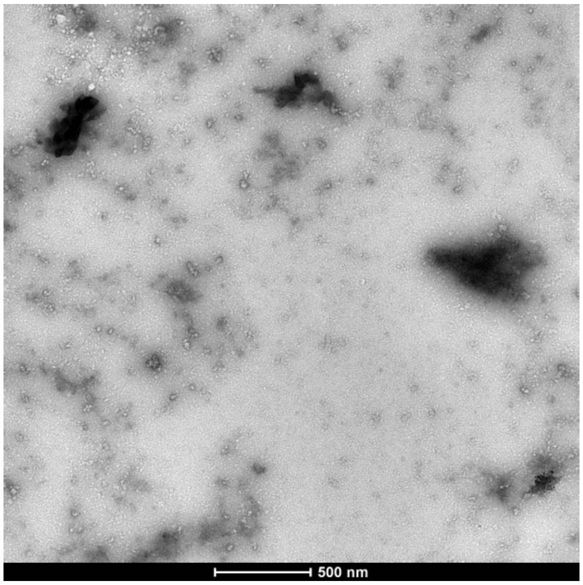

Figure S6 – Negative stain TEM of the sample only M85 protein preparation after its aggregation at 37 °C for 4 days.

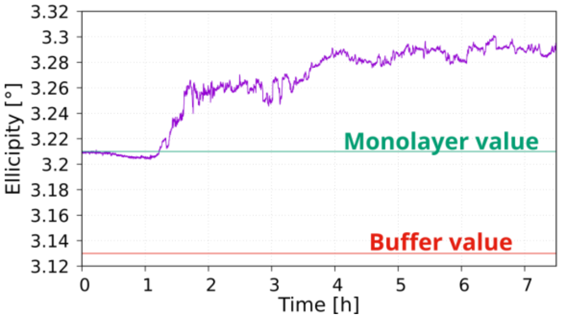

Figure S7 – Ellipsometry analysis on the M85 – lipid monolayer interaction kinetics. The insertion of material can be observed by the increase of ellipticity, leading to an increase of the surface excess on the air/water interface. The lag time is more likely due to the time required by the protein to diffuse and interact with the lipid monolayer.

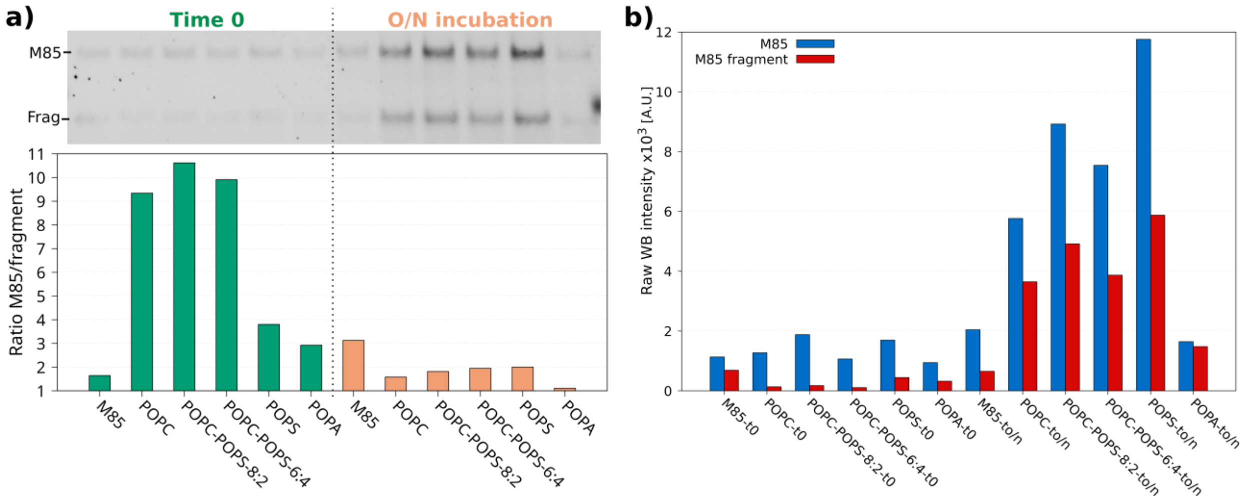

Figure S8 – Western blot addressing the time course interaction of the two M85 fragments present in the final M85 preparation with different composition LUVs. The graph in panel a represents the ratio between M85 full length and its fragment. As soon as the M85 preparation was added into the LUVs samples, the "Time 0" aliquots were taken. The LUVs from the samples were isolated by centrifugation and analyzed by western blot. M85 full length appears to bind lipid membranes faster than its fragment, since the ratio is higher at time 0. After the O/N incubation, the fragment is also associated with the membrane. Hypothetically, the C-terminal domain (CTD) has an importance in the interaction, because it is the only portion that lacks in the fragment compared to the M85 full length. Therefore, CTD might stabilize this interaction. Panel b represents the raw data extracted from the western blot.

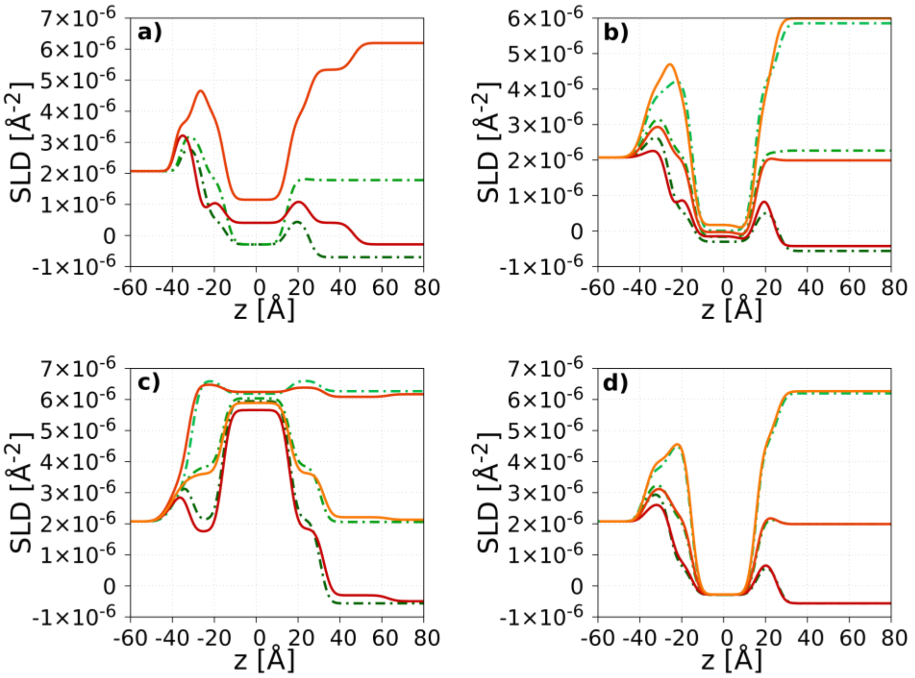

Figure S9 –SLD profiles from the neutron reflectivity analysis, in green colors the SLD profiles for the sample before the M85 mixture injection and in red colors after the injection, incubation and cell wash. a) POPC SLB, b) POPC:POPS 8:2 SLB, c) Dpol SLB and d) POPC:POPA 8:2.

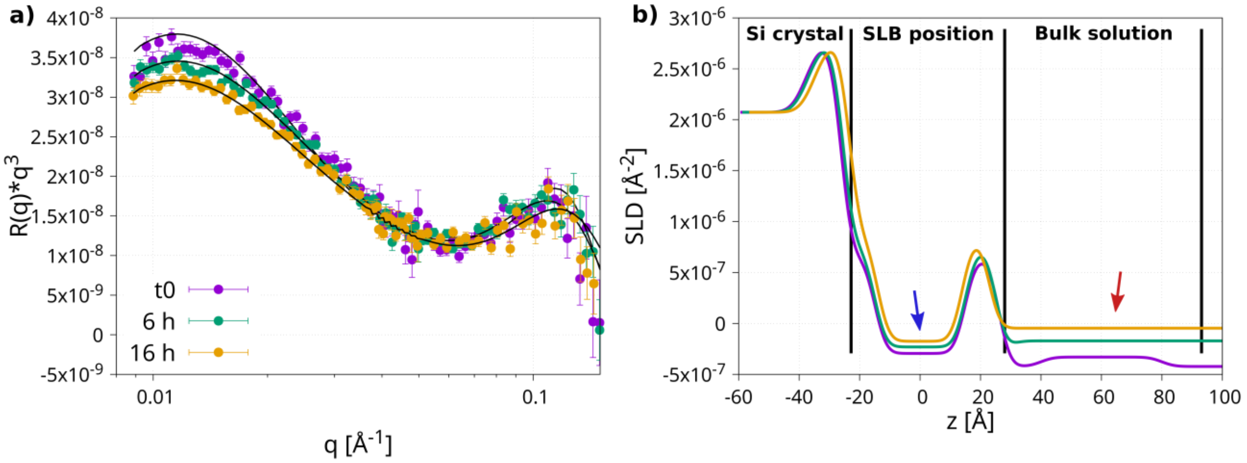

Figure S10 – Additional neutron reflectivity kinetics analysis carried out on the sample POPC:POPS 8:2. a) is the neutron reflectivity plotted along with their theoretical reflectivity profiles and in b) is displayed the minimized SLD profile. It can be noticed as at "t0" the M85 is placed outside the lipid bilayer (red arrow). Upon time, the SLD of the center of the bilayer started to increase (blue arrow) as well as the SLD of the external part. This leads to an insertion upon time of M85 into the membrane, as well as aggregation formation above the membrane. In this

case, the area per lipid was a fit parameter, since the insertion of the protein into the SLB might cause a change in the stiffness of the membrane.
